# Artery Wall Assessment Helps Predict Kidney Transplant Outcome

**DOI:** 10.1371/journal.pone.0129083

**Published:** 2015-06-12

**Authors:** Domingo Hernández, Javier Triñanes, Eduardo Salido, Sergio Pitti, Margarita Rufino, José Manuel González-Posada, Armando Torres

**Affiliations:** 1 Nephrology Department, Carlos Haya Regional University Hospital and University of Málaga (IBIMA), REDinREN, Málaga, Spain; 2 Research Unit, Hospital Universitario de Canarias, Tenerife, Spain; 3 Radiology Department, Hospital Universitario de Canarias, Tenerife, Spain; 4 Nephrology Department, Hospital Universitario de Canarias, CIBICAN, University of La Laguna, Instituto Reina Sofía de Investigación Renal (IRSIN), Tenerife, Spain; The University of Tokyo, JAPAN

## Abstract

**Background:**

Kidney transplant recipients have high cardiovascular risk, and vascular inflammation may play an important role. We explored whether the inflammatory state in the vessel wall was related to carotid intima-media thickness (c-IMT) and patient survival following kidney transplantation.

**Methods:**

In this prospective observational cohort study we measured c-IMT and expression of proinflammatory cytokines and adhesion molecules in the inferior epigastric artery in 115 kidney transplant candidates. Another c-IMT measurement was done 1-year post-transplantation in 107. By stepwise multiple regression analysis we explored factors associated with baseline c-IMT and their changes over time. Multivariate Cox regression analysis was constructed to identify risk factors for mortality.

**Results:**

A worse cardiovascular profile (older age, smoker, diabetic, carotid plaque, systolic blood pressure and vascular calcification) and higher VCAM-1 levels were found in patients in the highest baseline c-IMT tertile, who also had a worse survival. Factors independently related to baseline c-IMT were age (β=0.369, *P*<0.0001), fasting glucose (β=0.168, *P*=0.045), smoking (β=0.228, *P*=0.003) and VCAM-1 levels (β=0.244, *P*=0.002). Independent factors associated with c-IMT measurement 1-year post-transplantation were baseline c-IMT (β=-0.677, *P*<0.0001), post-transplant diabetes (β=0.225, *P*=0.003) and triglycerides (β=0.302, *P*=0.023). Vascular VCAM-1 levels were associated with increased risk of mortality in bivariate and multivariate Cox regression. Notably, nearly 50% of patients showed an increase or maintenance of high c-IMT 1 year post-transplantation and these patients experienced a higher mortality (13 versus 3.5%; *P*=0.021).

**Conclusion:**

A worse cardiovascular profile and a higher vascular VCAM-1 protein levels at time of KT are related to subclinical atheromatosis. This could lead to a higher post-transplant mortality. Pre-transplant c IMT, post-transplant diabetes and triglycerides at 1-year post-transplantation may condition a high c-IMT measurement post-transplantation, which may decrease patient survival.

## Introduction

Chronic kidney disease (CKD) is an independent risk factor for cardiovascular disease (CVD) and premature death, primarily due to acceleration of the atheromatosis process [1, 2]. Although traditional cardiovascular risk factors are highly prevalent in CKD patients, they do not completely explain the elevated risk, suggesting that other mechanisms may be crucial. Indeed, both endothelial dysfunction and vascular inflammation are processes involved in the development of atheromatosis in renal patients, especially when traditional and uremia-related risk factors concur [3–5].

Atheromatosis is a dynamic and complex inflammatory process of the artery wall. The endothelium overlying atherosclerotic lesions may express vascular cell adhesion molecule-1 (VCAM-1), intercellular adhesion molecule-1 (ICAM-1), the monocyte chemoattractant protein-1 (MCP-1) and interleukin-6 (IL-6), which have all been shown to be closely linked with infiltration and proliferation of atheromatosis-related inflammatory cells [6–8]. These soluble molecules are predictors of cardiovascular mortality in both CKD patients and kidney transplant (KT) recipients [3–5, 9, 10], but their expression in the artery wall has not been assessed. In addition, the relationship between the soluble inflammatory markers (including C-reactive protein) at the time of KT and the evolution of post-transplant atherosclerosis is controversial [11], suggesting that increased cardiovascular mortality in these patients may be explained by a higher pre-transplant atherosclerotic burden. We hypothesized, therefore, that artery wall assessment at the time of transplantation could better elucidate the pathophysiologic relationship between atheromatosis-associated pre-transplant inflammatory state and the evolution of post-transplant atherosclerosis, which could help to predict long-term survival.

Common carotid artery intima media thickness (c-IMT) is an early marker of subclinical atheromatosis, which correlates with an increased risk of CVD in both the general population and renal patients, including KT recipients [12, 13]. Nevertheless, few studies have examined the precise relationship between c-IMT measurements and pro-inflammatory cytokines and adhesion molecules involved in the atherogenic process of KT recipients, clinical setting where multiple classical and non-traditional risk factors are present. In addition, there are no conclusive data regarding the impact of changes over time in the c-IMT on adverse outcomes after KT [14–18].

This cohort study was performed to determine whether the in vivo expression levels of proinflammatory cytokines and adhesion molecules were associated with c-IMT at the time of KT. We also proposed to explore whether the inflammatory state in the vessel wall was related to c-IMT after the first post-transplant year and long-term patient survival following KT.

## Materials and Methods

### Participants and tissue samples

This prospective observational cohort study evaluated 148 consecutive adult CKD patients who received a deceased KT between August 2007 and April 2009 in a regional transplant center (Hospital Universitario de Canarias, Tenerife, Spain). We excluded 33 patients who were unable to undergo a perioperative carotid echography study. Thus, the final study population comprised 115 CKD patients who underwent a baseline echographic study at the time of KT. All the patients received conventional immunosuppression (steroids, tacrolimus and mycophenolate mofetil). During the first post-transplant year, 5 patients died and 3 experienced graft loss. Thus, 107 underwent a second carotid echography study 12 months after KT. Part of the methodology of this study has been reported previously [5]. Relevant clinical information about the donors and recipients was extracted prospectively from the Canary Islands Renal Transplant database, which has been updated yearly since 1996 [19]. The study was purely descriptive, and no attempts were made to modify any therapeutic aspect. Medical record review was performed according to the Spanish law.

The study was approved by the ethics committee of the Hospital Universitario de Canarias (Tenerife, Spain) and conducted following the Declaration of Helsinki and Istanbul. Written informed consent was obtained from all patients.

During surgery, a sample of the inferior epigastric artery (IEA) was obtained from all the participants. Tissue was quickly dissected in three sections for the different analyses: gene expression, protein quantification and histological analysis. Artery segments were fixed, paraffin-embedded and processed as previously reported [5]. Briefly, the reduction in arterial lumen was calculated as the percentage of lumen lost from the total lumen area measured at the inner elastic lamina, and the fibrosis as the proportion of the total area, including media and intima layers, positive for Sirius red staining. MCP-1, IL-6, ICAM-1 and VCAM-1 expression was ascertained by immunohistochemistry on serial sections as described [5]. All images were taken with a microscope Olympus DX41 (Tokyo, Japan) fitted with a Canon DP72 camera (Tokyo, Japan), and then analyzed using the software ImageJ (NIH) and the plug-in WCIF from Western Research Institute (Toronto, Canada).

The relative mRNA abundance was quantified using quantitative PCR (qPCR) and the SYBR Green detection method, as described [5], using total RNA isolated from EIA fragments similar to those used for immunohistochemistry. Real-time monitoring of the amplification process was performed with the iQ5 system (Bio-Rad, California, USA) and expression of each gene was normalized with the reference genes (18S rRNA and RNase P). The data were analyzed using the qBASE software [20].

Finally, another fragment of EIA was lysed in RIPA buffer and the quantification of IL-6, MCP-1, ICAM-1 and VCAM-1 was determined by using xMAP technology, as described [5]. The concentration of each analyte was determined for each sample and then corrected for the total protein concentration; the result obtained is expressed as picograms of analyte per microgram of total protein.

### c-IMT measurements

The baseline c-IMT was determined within 2 weeks after surgery by an experienced radiologist (SP) blinded to clinical data according to the standard clinical procedure [13], using a SSA-380 ultrasound transducer (Toshiba, Tokyo, Japan). Either an L6-7 MHz or a linear array transducer was used depending on the artery depth. Measurements of the left and the right common carotid were obtained in both the sagittal and axial insonation planes. The highest thickened diffuse point without plaques was selected for measurement and the mean value was used for analysis. A second carotid echographic study was performed 12 months after KT by the same radiologist. A low intraobserver variability has previously been reported in our radiology section after repeated measurements (mean intraclass concordance correlation 0.96 [95% CI 0.90–0.99]; *P*<0.001) [13]. Lastly, the presence of carotid plaques was also recorded.

### Outcome

All-cause mortality and graft loss were recorded. The last follow-up was on December 31, 2013. Survival was measured in months from the date of transplantation.

### Statistical analysis

**Fig 1:**
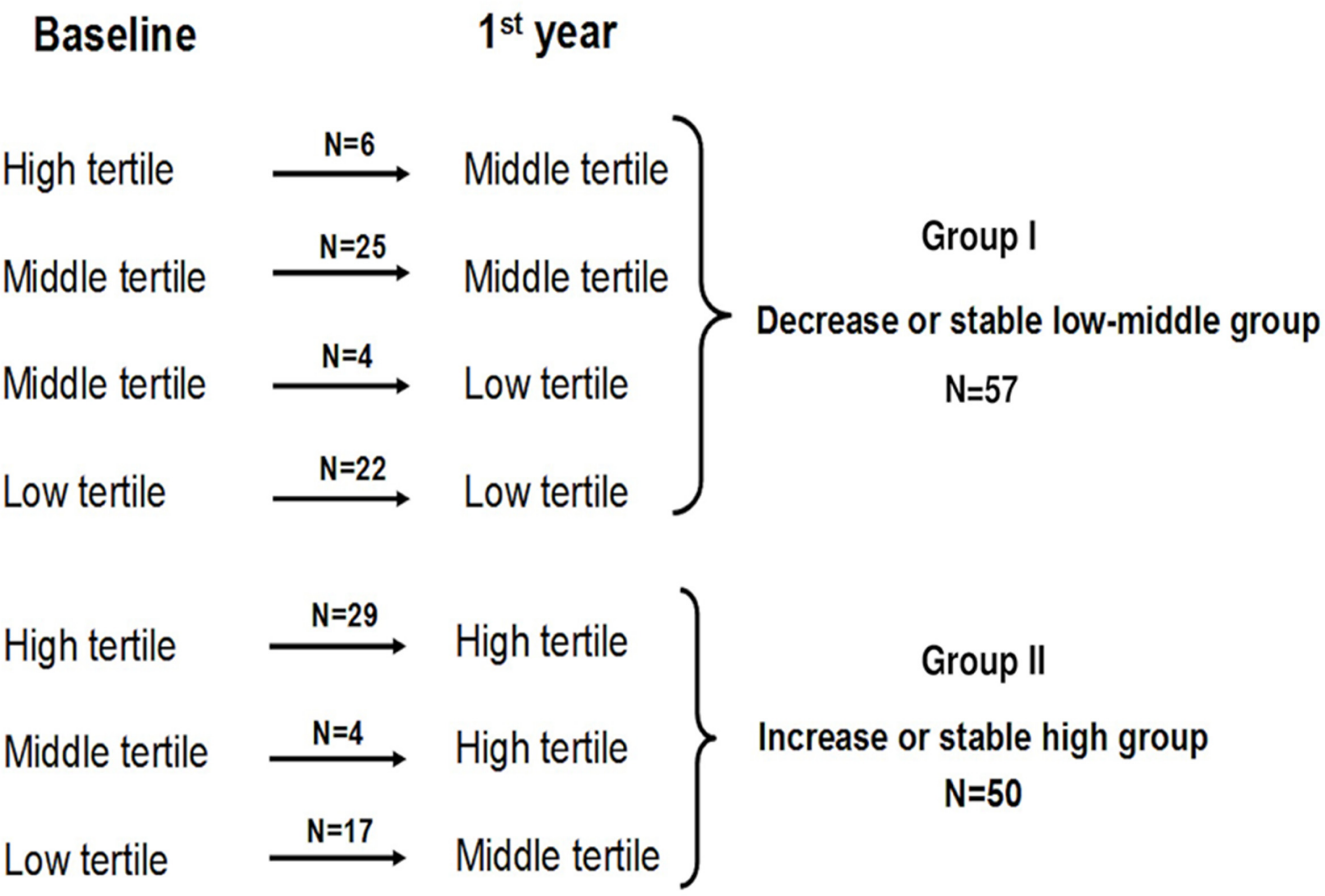
Variation patterns of c-IMT tertiles according to the fluctuation between the c-IMT measurements during study. No patients with a high baseline c-IMT evolved to a low c-IMT tertile at the first year post-transplantation. Similarly, no patients with a low baseline c-IMT tertile evolved to a high c-IMT tertile.

Baseline c-IMT measurements were divided into tertiles in order to assess the clinical characteristics. After the second echographic study, patients were clustered in two groups (from the nine possible combinations) according to the fluctuation between the c-IMT tertiles at both time points (Fig 1). Group I: individuals who changed to a lower tertile or who maintained both values within the lower or the middle tertile were classified as “decrease or stable low-middle stable”; and Group II: individuals who increased to the next tertile or with both values within the highest tertile were labeled as “increase or high stable”. Data are expressed as mean±SD (normally distributed data), median and interquartile range (non-normally distributed data) or as percent frequencies. Inter-group comparisons of quantitative variables were made by Student *t* test or the Mann-Whitney U-test as appropriate. ANOVA (or the Kruskal-Wallis test as appropriate) was used to compare continuous variables between different tertiles. The Bonferroni procedure was used for multiple comparisons of equivalence. Categorical data were compared using the chi-square test and Fisher exact test as appropriate. Simple correlations were explored with the Spearman coefficient. Stepwise multiple regression analysis was performed to determine independent factors associated with both baseline and final c-IMT measurements. The Kaplan-Meier test was used for survival analysis. Univariate, bivariate (models adjusting for other risk factors considered one by one) and multivariate Cox regression (entering risk factors two by two) were performed to identify risk factors for mortality. Calculations were made using the SPSS statistical package 15.0 (SPSS, Chicago, IL). *P* values<0.05 were considered significant.

## Results

### Baseline c-IMT measurements and clinical characteristics

Table 1 shows the clinical characteristics and biochemical data for the different baseline c-IMT tertiles. As expected, age, smoking, and proportion of diabetics were significantly higher in the highest c-IMT tertile. Although a good blood pressure control was achieved in the c-IMT tertiles, systolic blood pressure was nevertheless significantly higher in the highest tertile. Accordingly, fasting glucose and HbA1c levels, as well as the number of patients with major vascular calcifications and carotid plaque, were increased significantly in the highest c-IMT tertile. In addition, fasting glucose levels correlated with baseline c-IMT measurements (*rho* = 0.47; *P*<0.0001) (S1 Fig). Finally, baseline c-IMT measurements correlated with the presence of baseline carotid plaques (*rho* = 0.354; *P*<0.0001). No significant differences were found between the different tertiles in other clinical parameters, including the use of statins, aspirin, beta-blockers, and renin-angiotensin system blockers (Table 1).

**Table 1:** Baseline clinical and pathological data of the artery wall in kidney transplant patients according to carotid intima-media thickness tertiles.

	T1 (N = 39)	T2 (N = 38)	T3 (N = 38)	*P* value
c-IMT tertile (mm)	<0.50	0.50 to 0.70	>0.70	-
Mean c-IMT (mm)	0.41±0.05	0.6±0.05^b^	0.86±0.1^a^	0.000
Carotid plaques (%)	8.3	23	68	0.000
Age (years)	38±11	50±12^b^	56±8^c^	0.000
Male (%)	67	66	79	0.371
Type of dialysis (% hemodialysis)	67	89	84	0.054
Pretransplant cardiovascular disease (%)	5	16	18.4	0.185
**Cause of CKD (%)**				0.019
*Diabetes*	7.7	18	34	
*Glomerulonephritis*	23	16	10.5	
*Polycystic kidney disease*	13	31	10.5	
*Nephroangiosclerosis*	2.6	-	2.6	
*Other*	51	24	37	
Body mass index (kg/m^2^)	24.4±4	26±4	26.2±5	0.163
Large vascular calcifications (%)	10	35	42	0.005
Time on dialysis (mo)	22±31	30±31	27±26	0.469
Use of ACEI/ARA (%)	15	10.5	10.5	0.752
Use of aspirin (%)	23	30	16	0.493
Use of statins (%)	15.4	24	21	0.648
Smokers (%)	18	26	50	0.007
Fasting glucose (mg/dL)	98±14	120±38^d^	131±42^b^	0.000
HbA1c (%)	5.3±0.7	6.1±1.1	6.3±1.1^e^	0.006
Hypertension (%)	95	92	95	0.859
Systolic blood pressure (mmHg)	130±18	134±17	141±14^f^	0.023
Diastolic blood pressure (mmHg)	73±5	75±9	77±9	0.189
Total cholesterol (mg/dL)	135±34	132±32	138±36	0.718
HDL-cholesterol (mg/dL)	41±12	42.5±15	39±12	0.747
LDL-cholesterol (mg/dL)	75±31	66±26	59±25	0.220
Triglycerides (mg/dL)	139±67	142±65	149±57	0.766
Media layer calcification (%)	22.6	47	70	0.001
Degree of fibrosis in the intima (%)	0.52±0.2	0.50±0.2	0.55±0.2	0.637
Degree of lumen reduction of IEA (%)	5.4±10	10.8±13	18±19^g^	0.001
**Death** (%)	2.6	13.2	23.7	0.023
*Cardiovascular*, *n*	-	2	6	
*Infection*, *n*	1	1	2	
*Neoplasia*, *n*	-	1	1	
*Other*, *n*	-	1	-	
Death-censored graft failure (%)	7.7	13	10.5	0.735

Abbreviations: c-IMT, carotid intima-media thickness; CKD, chronic kidney disease; ACEI/ARA, angiotensin-converting enzyme inhibitor/angiotensin receptor antagonist; IEA, inferior epigastric artery.

^a^ *P*<0.0001 vs. T2 and T1;

^b^ *P*<0.0001 vs. T1;

^c^ *P* = 0.033 vs T2;

^d^ *P* = 0.012 vs. T1;

^e^ *P* = 0.006 vs. T1;

^f^ *P* = 0.023 vs. T1;

^g^ *P* = 0.001

### Artery wall pathological changes

Overall histopathological analysis showed a greater degree of lumen reduction in the IEA among patients in the highest c-IMT tertile (Table 1), and this luminal narrowing was age dependent (*rho* = 0.34, *P* = 0.004). A trend toward a greater proportion of fibrosis in the intima was observed in the highest c-IMT tertile. Additionally, c-IMT measurements correlated with the degree of arterial lumen narrowing (*rho* = 0.416, *P*<0.0001). As previously reported [5], the intimal thickening for the IEA was mostly composed of smooth muscle actin (SMA)-positive cells and collagen fibers. No intimal calcification was observed in any IEA sample, while a higher incidence and severity of calcification in the media layer was present in the highest c-IMT tertile (Table 1). Finally, medial calcification was significantly associated with age (*rho* = 0.46, *P* = 0.001) and fasting glucose (*rho* = 0.55, *P*<0.0001) in all the patients. After excluding diabetic patients, only age showed a correlation with medial calcification (*rho* = 0.39, *P* = 0.001).

### Proinflammatory cytokines, adhesion molecules and c-IMT

**Fig 2:**
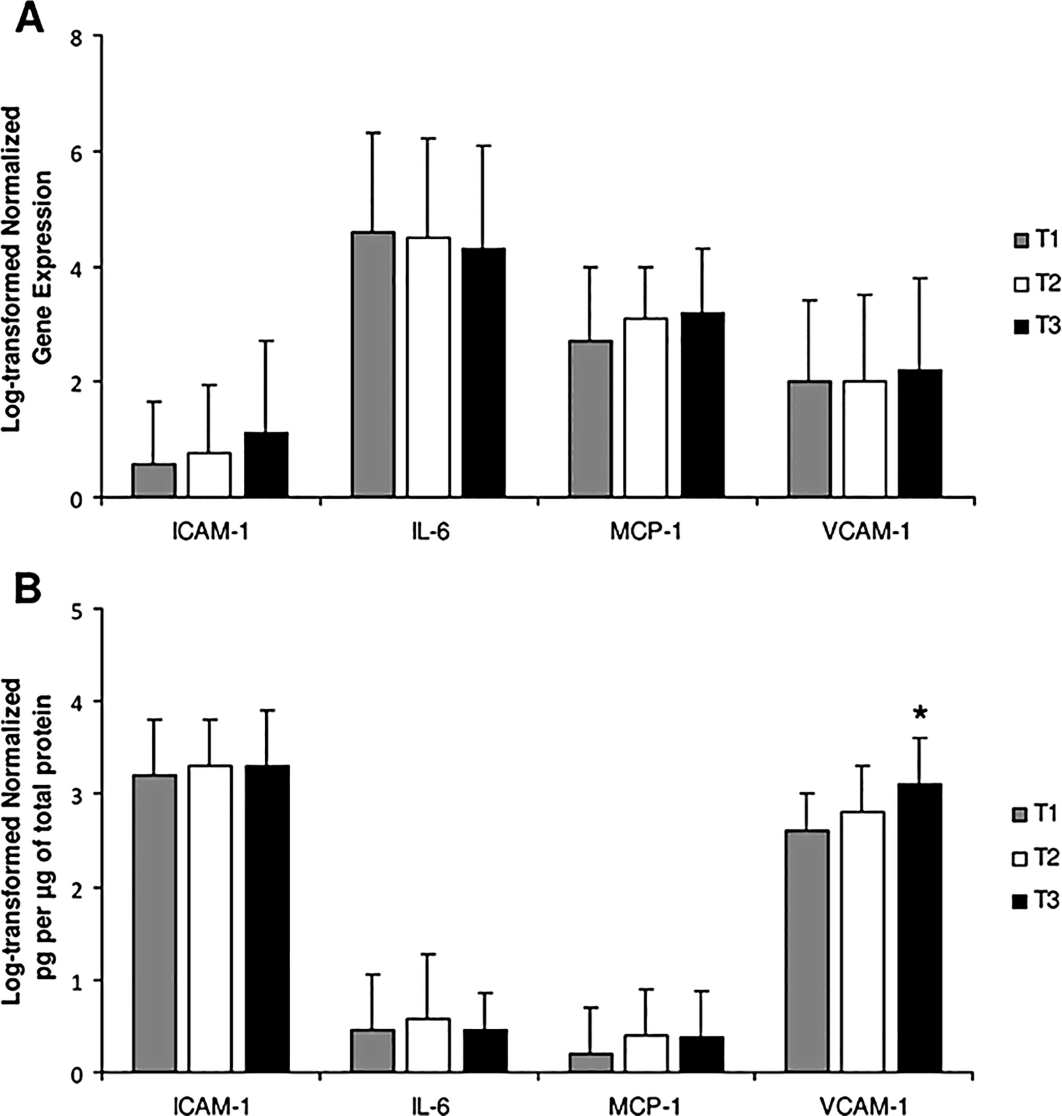
Proinflammatory cytokines, adhesion molecules and c-IMT measurements. A) Differences in the gene expression of proinflammatory markers in the artery wall according to c-IMT tertiles. B) Differences in the quantification of proinflammatory proteins by c-IMT tertiles. *ANOVA test for VCAM-1, *P* = 0.003; Bonferroni procedure, T3 vs. T1, p = 0.003; T3 vs T2, *P* = 0.076.

Although a trend toward a higher mRNA expression of ICAM-1 in the IEA was only observed in the highest c-IMT tertile, the VCAM-1 protein levels were significantly increased in the highest c-IMT tertile compared with the rest (Fig 2). No significant differences were observed for the expression of IL-6 or MCP-1 between tertiles. Interestingly, when vascular VCAM-1 protein levels were divided into tertiles, age, c-IMT measurements, as well as a major proportion of cardiovascular disease and carotid plaques disease were significantly increased in the highest VCAM-1 tertile (Table 2). Likewise, a major degree of arterial lumen reduction was observed among patients in the highest VCAM-1 tertile, and this luminal narrowing correlated with the vascular VCAM-1 protein levels (*rho* = 0.339, *P*<0.0001). Accordingly, VCAM-1 protein levels correlated with both baseline c-IMT measurements (*rho* = 0.380, *P*<0.0001) (S1 Fig) and the presence of baseline carotid plaques (*rho* = 0.339, *P*<0.0001). A similar correlation was also observed after excluding diabetic patients. Finally, VCAM-1 protein levels were significantly higher in patients with baseline carotid plaques compared with the rest (3.1±0.4 vs. 2.7±0.4 log pg/μg of total protein; *P*<0.0001).

**Table 2:** Baseline clinical and pathological data of the artery wall in kidney transplant candidates according to VCAM-1 protein level tertiles.

	T1 (N = 39)	T2 (N = 38)	T3 (N = 38)	*P* value
Log VCAM-1 tertile (pg/μg)	<2.5	2.5 to 3.1	>3.1	-
Mean log VCAM-1 (pg/μg)	2.1±0.2	2.8±0.2^b^	3.4±0.2^a^	0.000
Mean c-IMT (mm)	0.54±0.2	0.58±0.2^d^	0.71±0.2^c^	0.001
Carotid plaques (%)	17	24	54	0.003
Age (years)	45.3±15	45.4±12^f^	53±9^e^	0.021
Male (%)	69	64	75	0.593
Type of dialysis (% hemodialysis)	69	85	89	0.094
Pretransplant cardiovascular disease (%)	6	8	28	0.014
**Cause of CKD** (%)				0.227
*Diabetes*	6	25	28	
*Glomerulonephritis*	28	7	14	
*Polycystic kidney disease*	25	20	11	
*Nephroangiosclerosis*	3	5	8	
*Other*	37	38	36	
Body mass index (kg/m^2^)	25.8±4	25.7±5	24.7±5	0.568
Large vascular calcifications (%)	22	24	44	0.051
Time on dialysis (mo)	23±34	28±30	30±27	0.642
Use of ACEI/ARA (%)	15	13	11	0.858
Use of aspirin (%)	17	14	36	0.158
Use of statins (%)	12	15	33	0.064
Smokers (%)	28	28	31	0.967
Fasting glucose (mg/dL)	108±37	118±38	124±35	0.182
HbA1c (%)	5.4±0.7	5.9±0.1	6.1±1.1	0.166
Hypertension (%)	97	92	92	0.643
Systolic blood pressure (mmHg)	132±21	133±14	139±16	0.195
Diastolic blood pressure (mmHg)	72±7	77±8	77±9	0.055
Total cholesterol (mg/dL)	138±35	136±36	135±32	0.963
HDL-cholesterol (mg/dL)	44±16	42±13	39±11	0.502
LDL-cholesterol (mg/dL)	75±38	66±28	63±23	0.483
Triglycerides (mg/dL)	118±54	159±77^g^	147±45	0.019
Media layer calcification (%)	38	48	62	0.152
Degree of fibrosis in the intima (%)	0.52±0.2	0.54±0.2	0.53±0.1	0.856
Degree of lumen reduction of IEA (%)	4.3±7	9.1±10^i^	21±20^h^	0.000
**Death** (%)	5	9	27	0.088
*Cardiovascular*, *n*	2	1	5	
*Infection*, *n*	-	1	3	
*Neoplasia*, *n*	-	1	1	
*Other*, *n*	-	-	1	
Death-censored graft failure (%)	11	14	7	0.551

Abbreviations: c-IMT, carotid intima-media thickness; CKD, chronic kidney disease; ACEI/ARA, angiotensin-converting enzyme inhibitor/angiotensin receptor antagonist; IEA, inferior epigastric artery.

^a^ *P*<0.0001 vs. T2 and T1;

^b^ *P*<0.0001 vs. T1 and T3;

^c^ *P* = 0.001 vs. T1;

^d^ *P* = 0.010 vs. T3;

^e^ *P* = 0.042 vs. T2;

^f^ *P* = 0.054 vs. T1;

^g^ *P* = 0.016 vs. T1;

^h^ *P*<0.0001 vs. T1;

^i^ *P* = 0.002 vs. T3.

By backward multiple regression analyses, age (standardized β = 0.369, *P*<0.0001), fasting glucose (standardized β = 0.168, *P* = 0.045), smoking (standardized β = 0.228, *P* = 0.003) and VCAM-1 protein levels (standardized β = 0.244, *P* = 0.002) were independently associated with baseline c-IMT. Overall, the model explained 41% of the c-IMT measurements. Importantly, when diabetic patients were excluded VCAM-1 protein levels maintained an independent association with baseline c-IMT (standardized β = 0.222, *P* = 0.013) after adjusting for confounders.

### Follow-up and outcome

After a median follow-up of 68 months (interquartile range 57–73) the overall mortality and death-censored graft failure rates were 13% and 10.4%, respectively. Patients in the highest c-IMT tertile showed a higher mortality rate compared with the middle and lower c-IMT tertiles (23.7 vs. 13.2 vs. 2.6%, respectively) (Table 1). Overall Kaplan-Meier survival estimates showed significant differences between c-IMT tertiles (log-rank analysis 7.3; *P* = 0.025) (S2 Fig). In addition, patients in the highest VCAM-1 tertile showed a trend toward a lower survival compared with the rest (77 vs. 89 vs. 93%, respectively) (log-rank analysis 4.8; *P* = 0.089) (S3 Fig). CVD was the leading cause of death (Table 1). By contrast, death-censored graft failure rates were comparable among study groups and chronic allograft failure was the main cause of graft failure in survivors.

Table 3 depicts the general clinical characteristics in the two groups according to the tertile variation after the second echographic study. Classical cardiovascular risk factors were more prevalent in Group II compared with Group I. Notably, new onset diabetes after transplantation (NODAT) within the first post-transplant year developed more frequently in Group II and fasting glucose at 1 year post-transplantation correlated with the final c-IMT (S1 Fig). Thus, triglyceride levels at the first post-transplant year were significantly higher in Group II. These patients had a greater proportion of intima-media fibrosis in the IEA (0.57±0.16 vs. 0.48±0.2; *P* = 0.034), media layer calcification (56 vs. 33%; *P* = 0.031) and a higher reduction of arterial lumen (14.5±18 vs. 6.6±8; *P* = 0.009) compared with Group I. A trend toward higher VCAM-1 protein levels was observed in Group II (2.9±0.4 vs. 2.7±0.4 log pg/μg; *P* = 0.096). No significant differences were observed in other clinical, pathological or inflammatory parameters between the groups. Again, VCAM-1 protein levels were only significantly correlated with the final c-IMT (S1 Fig). By backward multiple regression analysis, baseline c-IMT (standardized β = 0.742, *P*<0.0001), NODAT (standardized β = 0.186, *P* = 0.003) and triglycerides at the first year post-transplantation (standardized β = 0.148, *P* = 0.023) were independently associated with the final c-IMT measurement.

**Table 3:** Clinical characteristics of patients after stratification according to c-IMT measurement variation after second carotid echographic study.

	Group I (N = 57)	Group II (N = 50)	*P* value
	Decrease or low-middle stable group	Increase or high stable group	
c-IMT (mm)	0.52±0.1	0.79±0.2	0.000
Carotid plaques (%)	22	60	0.000
Age (years)	44.4±14	50.7±11	0.010
Male (%)	70	60	0.741
Pretransplant CVD (%)	11	12	0.863
Large vascular calcifications (%)	21	35	0.116
Diabetics prior to KT (%)	10.5	28	0.021
Time on dialysis (mo)	23±16	27±34	0.421
Use of ACEI/ARA (%)	16	9	0.337
Use of aspirin (%)	30	18.5	0.306
Use of statins (%)	21	19	0.816
Acute rejection (%)	25	18	0.449
Smokers (%)	23	39	0.094
NODAT (%)	5	20	0.036
Fasting glucose at 1^st^ year (mg/dL)	105±29	131±43	0.007
HbA1c at 1^st^ year (%)	5.8±1.2	5.9±1	0.948
Hypertension at 1^st^ year (%)	70	80	0.291
Systolic blood pressure (mmHg)	133±14	130±15	0.287
Diastolic blood pressure (mmHg)	75±11	73±7	0.304
Total cholesterol at 1^st^ year (mg/dL)	174±34	175±34	0.932
Triglycerides at 1^st^ year (mg/dL)	117±45	143±71	0.053
Serum creatinine at 1^st^ year (mg/dL)	1.3±0.4	1.2±0.3	0.786
Total proteinuria at 1^st^ y. (g/day)	0.2±0.3	0.25±0.3	0.593
Media layer calcification (%)	33	55.6	0.009
**Death** (%)	1.8	17.6	0.043
*Cardiovascular*, *n*	1	5	
*Infection*, *n*	1	2	
*Neoplasia*, *n*	-	1	
Death-censored graft failure (%)	7	9.8	0.734

Abbreviations: c-IMT, carotid intima-media thickness; CVD, cardiovascular disease; KT, kidney transplantation; ACEI/ARA, angiotensin-converting enzyme inhibitor/angiotensin receptor antagonist; NODAT, new onset diabetes after transplantation.

**Fig 3:**
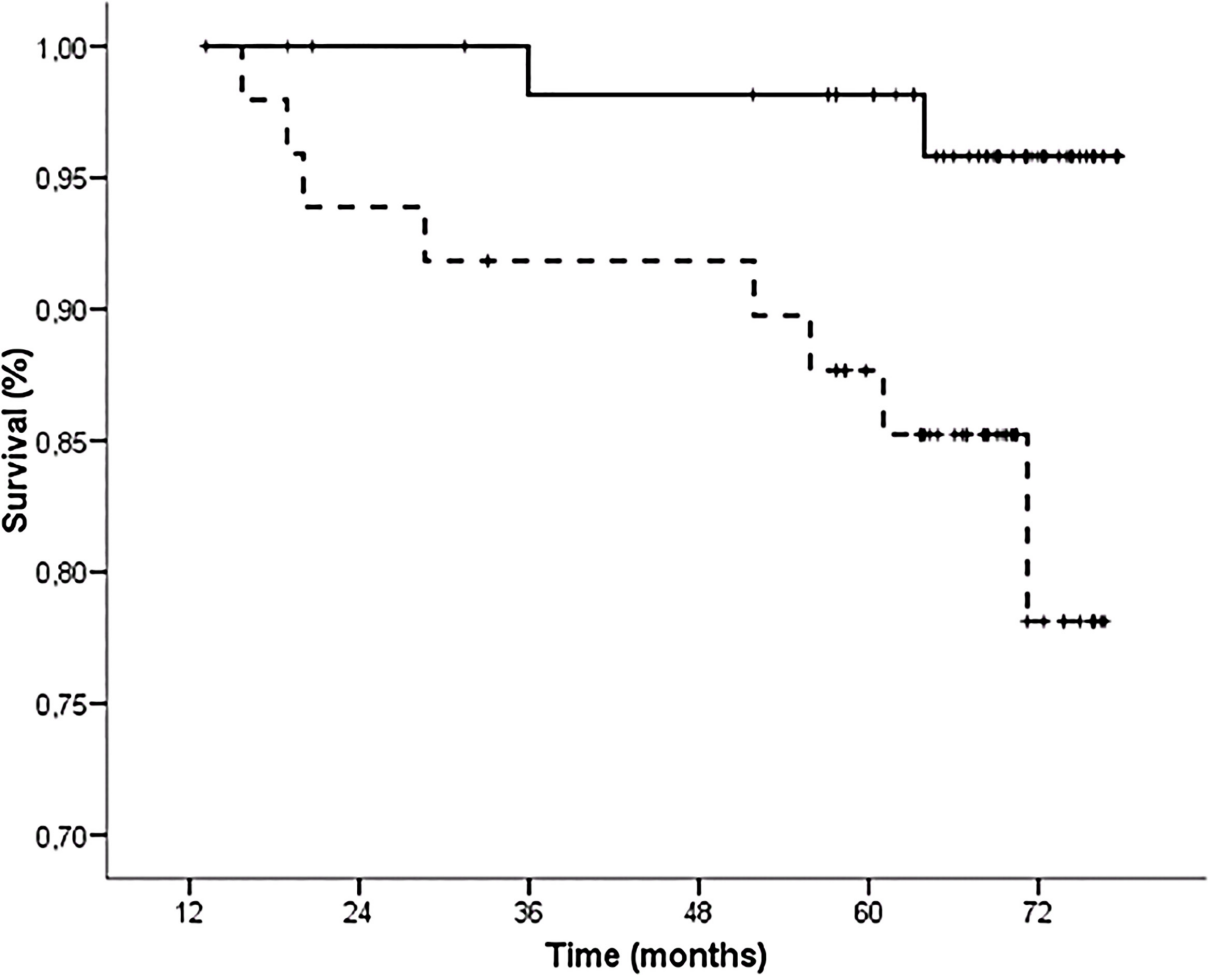
Kaplan-Meier curves according to variation patterns between the c-IMT tertiles at both time periods. Solid line indicates the “decrease or stable low-middle” group and dotted line the “increase or stable high” group (log-rank analysis 5.4; *P* = 0.021).

After the second echographic study, 10 patients died and 9 had graft failure. Kaplan-Meier estimates showed that Group II patients experienced a significantly higher mortality compared with Group I during the follow-up (Fig 3). Notably, higher VCAM-1 protein levels were observed in the patients who died during the follow-up compared with the survivors (3.2±0.5 vs. 2.7±0.4 log pg/μg; *P* = 0.003). Interestingly, bivariate Cox regression analysis showed that VCAM-1 protein levels were a strong predictor of death after adjustments for potential confounders, including both baseline and final c-IMT measurements (Table 4). Finally, age, time on dialysis and VCAM-1 protein levels also remained independently associated with mortality in multivariate Cox regression analysis entering all the risk factors considered in the bivariate analysis two by two (Table 4).

**Table 4:** Relationship between VCAM protein levels and overall mortality by bivariate and multivariate Cox regression analysis

Bivariate analysis			Multivariate analysis*		
Adjusted for	Hazard ratio (95% CI)	*P* value	Variables	Hazard ratio (95% CI)	*P* value
Age	4.5 (1.3–15)	0.015	Age	1.07 (1.01–1.14)	0.038
Gender	5.5 (1.6–18)	0.006	Time on dialysis	1.02 (1.01–1.03)	0.004
Body mass index	5.9 (1.7–20)	0.004	Log VCAM-1	6.6 (1.5–30)	0.019
Smoking	6.1 (1.7–22)	0.005			
Baseline c-IMT	3.5 (1.01–13)	0.050			
Highest baseline c-IMT tertile	4.2 (1.1–15)	0.033			
Final c-IMT	4.5 (1.2–16)	0.024			
Highest final c-IMT tertile	4 (1.1–14)	0.028			
Pretransplant diabetes	5.6 (1.6–19)	0.007			
Pretransplant CVD	4.5 (1.3–15)	0.014			
Baseline systolic blood pressure	6.5 (1.5–27)	0.011			
Time on dialysis	6.1 (1.7–21)	0.006			
Hemodialysis (vs. PD)	5.2 (1.5–18)	0.011			
Large VC	3.6 (1.2–11)	0.025			
Carotid plaques	4.7 (1.1–20)	0.034			
NODAT	6.8 (1.9–25)	0.003			
Baseline T-cholesterol	5.9 (1.7–20)	0.005			
Group II (vs. Group I)	4.9 (1.1–23)	0.045			

Abbreviations: c-IMT, carotid intima-media thickness; KT, kidney transplantation; VC, vascular calcifications; NODAT, new onset diabetes after transplantation; PD, peritoneal dialysis; T-cholesterol, total cholesterol. Group II, patients who showed an increase to the highest tertile or who maintained both values within the highest tertile; Group I, patients who showed a reduction to a lower tertile or who maintained both values within the lower or the middle tertile.

*Because the number of events was few, this analysis was performed entering risk factors two by two.

## Discussion

This study shows that, in the presence of both traditional and uremia-related risk factors, VCAM-1 production in the IEA may be a marker for the development of more severe atheromatous lesions and a higher c-IMT in unselected KT candidates. Certainly, we cannot prove a causal role of VCAM-1 for atherosclerosis in this particular population. However, our data provide light on a pathogenic mechanism involved in the inflammation-related atheromatosis process at the artery wall of these patients, which could be a relevant predictor of survival following KT. To our knowledge, this is the first study designed to elucidate the impact of the production of VCAM-1 in the IEA on both c-IMT measurements and survival in KT recipients with different degrees of subclinical atheromatosis at transplantation. Furthermore, ongoing changes in the c-IMT 12 months after KT provided prognostic clinical information. The mean c-IMT of our study population was similar to that of other Caucasian populations, as was the distribution of c-IMT in tertiles [14, 21].

Inflammation-related endothelial dysfunction is associated with CVD, including subclinical atheromatosis, in the general population and renal patients [3, 22]. However, the precise relationship between c-IMT measurements and the vascular generation of molecules involved in the pathogenesis of atheromatosis of unselected KT candidates has not been studied. Interestingly, we found a significantly higher expression of VCAM-1 in the IEA of patients in the highest c-IMT tertile. Accordingly, c-IMT measurements correlated with VCAM-1 levels and the degree of arterial lumen reduction. Importantly, VCAM-1 protein levels were independently associated with baseline c-IMT in multiple regression analysis, suggesting that this parameter may be an early surrogate marker of atheromatosis in KT candidates, mainly when other risk factors concur. Indeed, risk factors for atherosclerosis such as age, diabetes, smoking and vascular calcifications were overrepresented among recipients in the highest c-IMT tertile. In agreement with our findings, previous studies have reported an independent significantly association between adhesion molecules and c-IMT measurements in other inflammatory states, including CKD [5, 23–25], corroborating the role of these molecules in the development of atherosclerosis-associated endothelial activation.

Although no significantly higher expression of IL-6 and MCP-1 was observed among patients in the highest c-IMT tertile, we cannot rule out the role of other proinflammatory cytokines for the development of atheromatosis in renal patients [5, 17, 26]. Indeed, we also found a strong correlation between all the inflammatory markers measured in the artery wall (data not shown), but VCAM-1 expression stood out. Thus, a pathogenic mechanism accounting for the role of adhesion molecules, especially VCAM-1, in renal patients seems plausible. In the presence of uremia, the clustering of risk factors leads to endothelial activation and the release of pro-inflammatory cytokines in the artery wall, initiating the mechanisms leading to the development of atherosclerosis. Cytokine activation produces dramatic up-regulation of adhesion molecule expression on the cell surface by SMCs resident in the intima layer with a synthetic phenotype, thus participating in cell-to-cell interaction and the recruitment of inflammatory cells [27]. Finally, activation of these inflammatory molecules induces the generation of fibrosis as the result of a final common pathway, perpetuating the inflammatory environment in the atherosclerotic lesion [28]. The fact that production of VCAM-1 correlated with more severe vascular lesions, e.g. increased arterial narrowing or more carotid plaques in the presence of abundant SMA-positive cells and collagen fibers in the intima layer, supports the role of VCAM-1 as a major marker of atherosclerosis, and that it is also necessary for neointima formation due to SMC migration [29, 30]. A plausible explanation for the correlation between VCAM-1 levels and the severity of vascular lesions could involve the induction of SMC migration to the neointima by VCAM-1 and the switching from a contractile to a synthetic SMC phenotype, leading to more severe lesions and higher production of cytokines and adhesion molecules. Histological studies in animal models of atheromatosis have shown that ICAM-1 immunostaining is stronger in the endothelium and weaker in intimal cells than VCAM-1, staining of which is much more restricted to lesions and abundant in neointimal cells [31]. These differential requirements for VCAM-1 and ICAM-1 in the formation of atherosclerotic lesions may indicate different roles for these molecules, with VCAM-1 playing a dominant role at the site of the lesion [32]. In agreement with this view, the patients in the highest VCAM-1 tertile experienced a higher degree of lumen reduction of IEA, and this luminal narrowing correlated with the vascular VCAM-1 protein levels. This agrees with the major proportion of cardiovascular disease and carotid plaques observed in our patients in the highest VCAM-1 tertile. The fact that soluble VCAM-1, but not ICAM-1, remained the only predictor of c-IMT in hypertensive patients with peripheral vascular disease [33] supports this hypothesis. Indeed, when we assessed longitudinally the impact of inflammatory markers in the IEA on survival after KT, intravascular VCAM-1 protein levels were a strong predictor of death in our multivariate Cox analysis. Accordingly, VCAM-1 protein levels were significantly higher in our patients with carotid plaques, which are prognostic factors for CVD in CKD [12, 34]. Although elevated soluble VCAM-1 is associated with post-transplant mortality, its expression in the artery wall was not investigated [9, 10]. These findings suggest that this molecule is an essential mediator in atheromatosis-related endothelial activation and dysfunction after KT. In support of this view, suppression of VCAM-1 has been shown to protect against atherosclerosis in an animal model [30, 35].

Successful KT may not completely ameliorate subclinical atheromatosis, possibly reflecting significant pre-existing vascular abnormalities. Thus, an increased baseline c-IMT may be a limiting factor for regression of c-IMT after KT, mainly in the presence of risk factors for atheromatosis. Indeed, nearly 50% of our patients showed an increase or maintenance of high c-IMT 1-year post-KT and a worse cardiovascular profile was observed in these patients, mainly due to glucose metabolism disorders. Additionally, baseline c-IMT, NODAT and triglycerides were independently associated with post-transplant c-IMT. Accordingly, individuals with a persistently high or increased c-IMT experienced a higher mortality in the univariate analysis. These findings agree with previous studies [13, 14, 16], and underscore the enhanced cardiovascular risk in this population, especially due to immunosuppressant side effects. Both immunosuppression-related insulin resistance and hyperglycemia may accelerate the development of atherosclerosis by triggering inflammation pathways leading to increased mortality over time, as reported [36–38]. The fact that c-IMT improves after successful pancreas transplantation within 2 years of the procedure [39] supports this view.

We found no relationship between pro-inflammatory molecules and final c-IMT in either non-diabetic or diabetic patients. A reduction in endothelial biomarkers such as VCAM-1 has been shown in KT recipients, suggesting improvement of endothelial injury [40, 41], but endothelial function evaluated by brachial artery flow-mediated dilation was not assessed [3]. Whether reduction of inflammation could translate into improvement of post-transplant CVD remains unanswered.

Our study has limitations. We did not assess the plasma levels of the cytokines and adhesion molecules evaluated. Plasma cytokine levels may vary greatly under certain uremia-related clinical conditions while soluble cellular adhesion molecules, particularly VCAM-1, have been found to be markers of post-transplant mortality [9]. Given that activated endothelial cells may be a major source of soluble VCAM-1 by cleavage from the plasma membrane, direct assessment of both proinflammatory gene activation and protein secretion in the artery wall may better highlight the pathways of inflammatory-related atheromatosis at the site of lesion, especially in high-risk renal patients. We performed a rigorous and accurate molecular and histological analysis, which provide reliability to our results. No significant differences were observed in gene expression of proinflammatory markers, though there were differences in protein secretion. We can only speculate about the dissociation between VCAM-1 mRNA and protein levels. Differences in protein and mRNA half-lives as well as post-translational regulation could be involved. We also have to take into account biological mechanisms such as cytokine secretion and the action of sheddases on adhesion molecules, which release the proteins to the circulation and alter the amounts of proteins in the artery wall. In addition, we cannot rule out that other inflammatory molecules, especially C-reactive protein, not determined in this study may be involved in the development of atheromatosis, as reported in CKD patients [42]. This is certainly a limitation of our study and it deserves future research. In addition, we did not perform physiological measures of endothelial function to complement VCAM-1 measurements, which represents another limitation of our study. Also, histological findings in the IEA may not be generalizable to other arterial territories. Nevertheless, autopsies of elderly patients who died of noncardiac diseases have documented that coronary arteries and IEA undergo progressive narrowing with age as a result of atheromatous histological lesions [43, 44], as in our study. Finally, the relatively small sample means additional studies are needed to enhance understanding of atheromatosis-related inflammatory pathways in renal patients.

## Conclusions

A worse cardiovascular profile and a higher VCAM-1 protein levels in the artery wall are associated with subclinical atheromatosis in KT candidates. This could lead to increased post-transplant mortality. Successful KT does not fully normalize c-IMT post-transplantation. Because both pre-transplant c-IMT and NODAT may condition an increased c-IMT post-transplantation, strict metabolic control could improve survival after KT.

## Supporting Information

S1 Fig c-IMT measurements, fasting glucose levels and VCAM-1 protein levels. Relationship between c-IMT measurements, fasting glucose levels and VCAM-1 protein levels in the artery wall at baseline (A and B) and at the first post-transplant year (C and D).
(TIFF)

S2 Fig Overall Kaplan-Meier curves according to baseline c-IMT tertiles. Log-rank analysis 7.3, *P* = 0.025. Log-rank test for comparison of survival between c-IMT tertiles (T3 vs. T1, *P* = 0.006; T2 vs. T1, *P* = 0.068; T3 vs. T2, *P* = 0.309).
(TIFF)

S3 Fig Overall Kaplan-Meier curves according to VCAM-1 protein tertiles. Log-rank analysis 4.8, *P* = 0.089. Log-rank test for comparison of survival between VCAM-1 tertiles (T3 vs. T1, *P* = 0.035; T2 vs. T1, *P* = 0.202; T3 vs. T2, *P* = 0.415)
(TIFF)
