# Supplementary figures and images for: Artery Wall Assessment Helps Predict Kidney Transplant Outcome
Source: PLoS One. 2015 Jun 12;10(6):e0129083. doi: 10.1371/journal.pone.0129083 (PMC4466324; doi:10.1371/journal.pone.0129083)

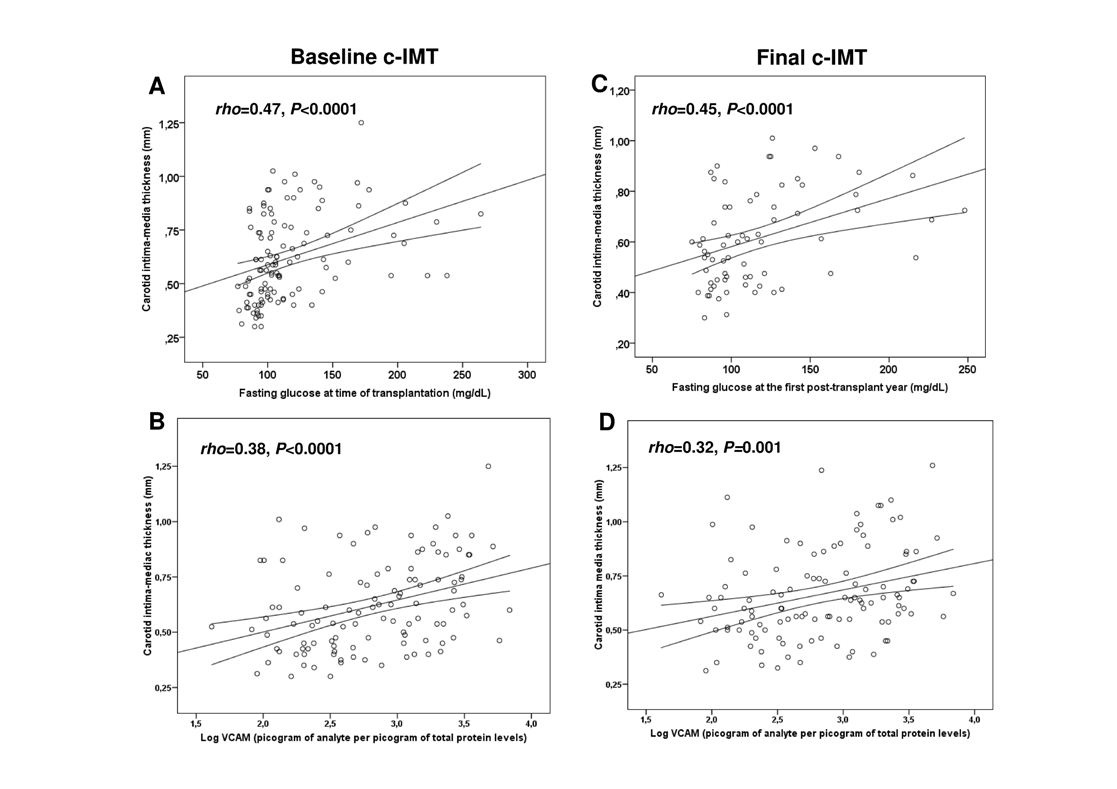

Supplement: S1 Fig — Relationship between c-IMT measurements, fasting glucose levels and VCAM-1 protein levels in the artery wall at baseline (A and B) and at the first post-transplant year (C and D). (TIFF) [file pone.0129083.s001.tiff]

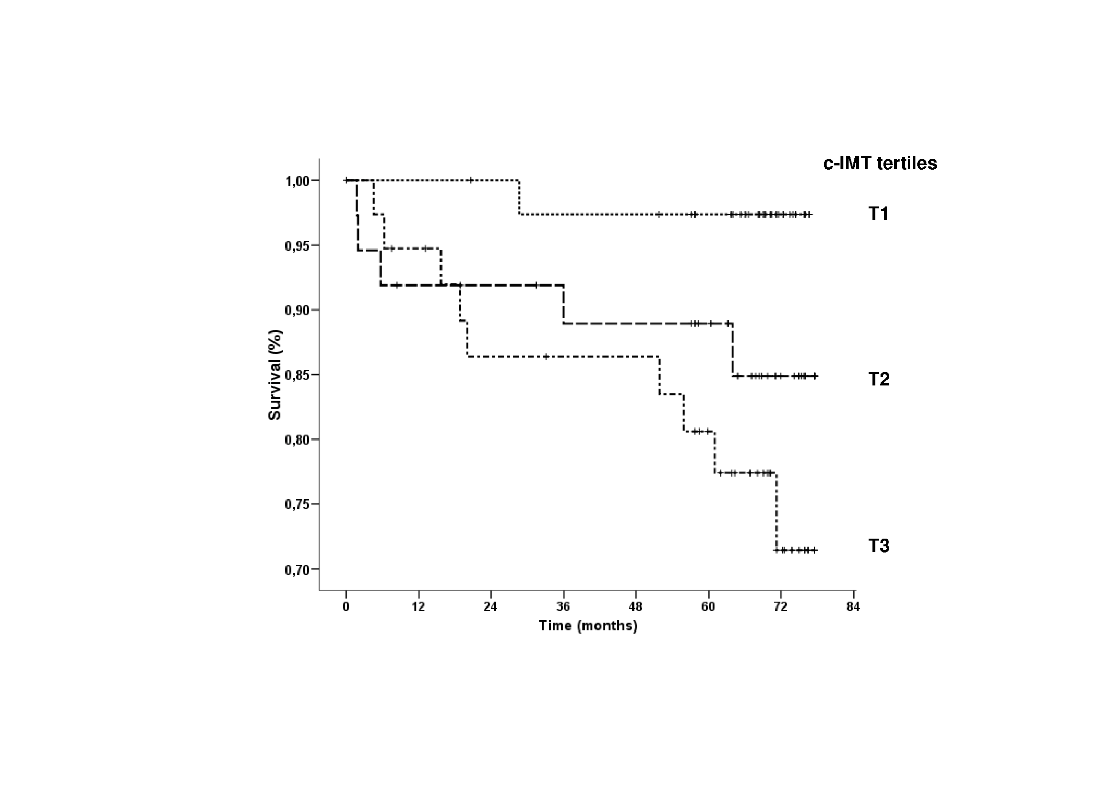

Supplement: S2 Fig — Log-rank analysis 7.3, P = 0.025. Log-rank test for comparison of survival between c-IMT tertiles (T3 vs. T1, P = 0.006; T2 vs. T1, P = 0.068; T3 vs. T2, P = 0.309). (TIFF) [file pone.0129083.s002.tiff]

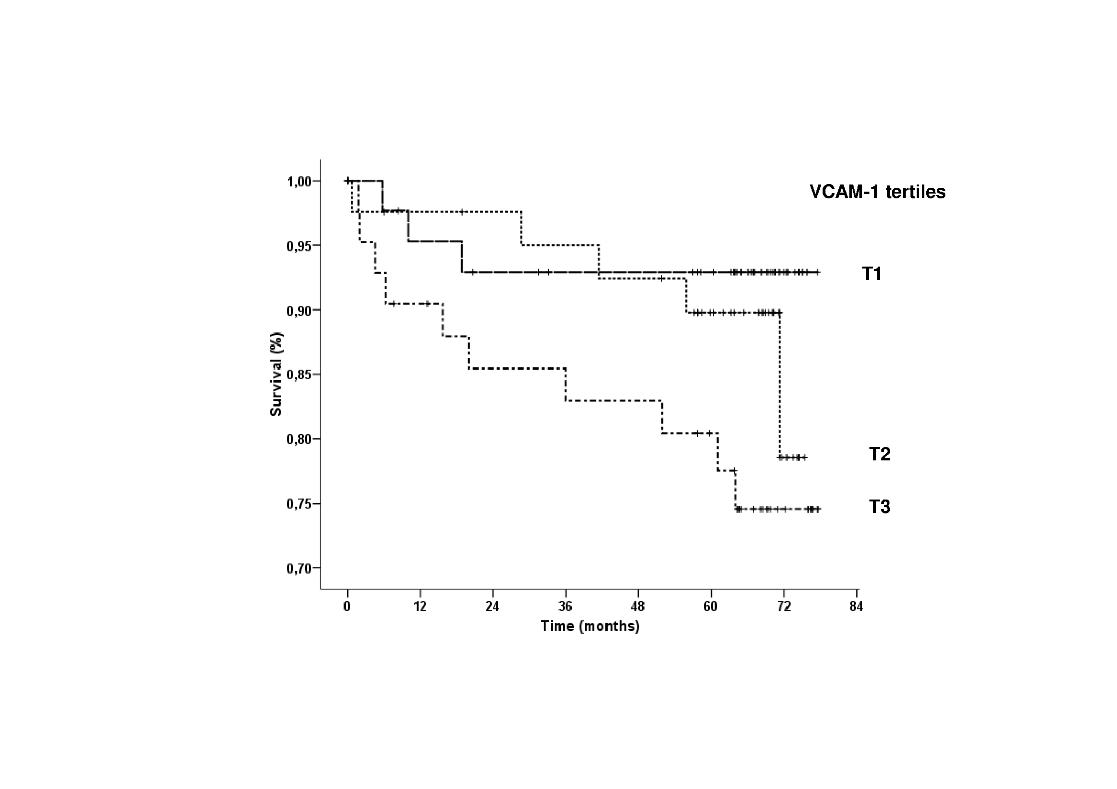

Supplement: S3 Fig — Log-rank analysis 4.8, P = 0.089. Log-rank test for comparison of survival between VCAM-1 tertiles (T3 vs. T1, P = 0.035; T2 vs. T1, P = 0.202; T3 vs. T2, P = 0.415) (TIFF) [file pone.0129083.s003.tiff]
